# Recognition of Depression and Help-Seeking Preference Among University Students in Singapore: An Evaluation of the Impact of Advancing Research to Eliminate Mental Illness Stigma an Education and Contact Intervention

**DOI:** 10.3389/fpsyt.2021.582730

**Published:** 2021-05-12

**Authors:** Gregory Tee Hng Tan, Shazana Shahwan, Edimansyah Abdin, Jue Hua Lau, Chong Min Janrius Goh, Wei Jie Ong, Ellaisha Samari, Kian Woon Kwok, Siow Ann Chong, Mythily Subramaniam

**Affiliations:** ^1^Research Division, Institute of Mental Health, Singapore, Singapore; ^2^President's Office, Nanyang Technological University, Singapore, Singapore

**Keywords:** mental illness, depression recognition, university students, help-seeking preference, anti-stigma intervention, knowledge-contact-based intervention

## Abstract

**Background:** The SMHS 2016 revealed that young adults in Singapore had the highest 12-month prevalence of mental disorders, with depression being the most prevalent condition. Additionally, the study found that those with higher education were less likely to seek treatment. The recognition of mental illness and knowledge of where to seek help has been found to influence one's ability to seek timely psychological help. This study thus aims to evaluate the effects of ARTEMIS, an education and contact intervention on university students' recognition of depression and help-seeking preference.

**Methods:** A total of 390 university students were recruited over a period of 6-months (October 2018 to April 2019). Students had to attend a one-off intervention which comprised a lecture on depression and personal contact with a person with lived experience of mental illness. Recognition of depression and help-seeking preference were assessed using a vignette approach, at pre- and post-intervention as well as at 3-month follow-up.

**Results:** The intervention was effective at improving student's recognition of depression and this effect was sustained at 3-months follow-up. The intervention was also effective in shifting student's help-seeking preference, although the effects were not sustained at 3-month follow-up. Having a close friend or family with mental illness was associated with better recognition, and being able to correctly recognize depression was linked to a preference to seek psychiatric over non-psychiatric help.

**Conclusion:** This study elucidated the efficacy of a knowledge-contact-based intervention in improving university students' recognition of depression and help-seeking preference. However, while the benefits on recognition of depression is more enduring, it is more transient for help-seeking beliefs, and booster sessions may be needed to improve the long-term effectiveness of the intervention on help-seeking preference. Lastly, to investigate the generalizability of this study's findings, future studies could replicate the current one across other non-self-selected samples, such as by integrating the intervention as part of student's orientation.

## Introduction

Mental health literacy (MHL) is defined by Jorm as “the knowledge and beliefs about mental illness which aids their recognition, management or prevention” (1). Research indicated that individuals with higher MHL are more likely to seek help than those with lower MHL (1–4). An important aspect of MHL is the recognition of mental illness, and corollary to a poor MHL is the failure to recognize and detect signs and symptoms of mental illness which has been found to lead to delayed help-seeking (5).

The knowledge of where to seek appropriate help from is another important component of MHL that influences one's ability to seek timely help from psychiatric professionals. Arguably, recognition of mental illness not only influences one's preference to seek help from mental health professionals (6), but also whether an individual would end up seeking treatment from inappropriate sources for their mental health issues, or delay seeking help from professional mental healthcare providers. In some Ugandan communities for instance, individuals tend to seek help from traditional healers for mental health issues, with conventional hospitals seen only as a last resort, as most of them have the tendency to believe that “they are bewitched” (7) rather than recognizing the symptoms as a sign of mental illness. In Malaysia, a study found that consulting *bomohs* (Malay Shaman or traditional medicine practitioner) was significantly higher in families that believed in supernatural causes of mental illness, with deep-rooted cultural beliefs cited as a major barrier to psychiatric treatment (8). A study among British South Asians had similar findings, where the majority of participants who believed in both supernatural and biological causes for psychosis followed the treatment prescribed by a faith healer and also took prescribed medications (9).

The reluctance to seek help or recommend help-seeking from psychiatric professionals is not uncommon in Singapore. The 2016 Singapore Mental Health Study (SMHS 2016) reported a significant 12-month treatment gap (78%) among individuals with mental disorders (10). Data from the SMHS2016 also elucidated some of the reasons cited by respondents who did not seek help which included “thinking the problem would get better by itself,” “being uncertain about where to go or who to see,” and “wanting to handle the problem on their own” (10). On a related note, a nationwide MHL study in Singapore found a preference among the lay public to recommend informal sources of help such as friends and family (11) for people with mental illness. However, friends and families may not always be able to recognize signs of mental illness or recommend help-seeking from mental health professionals, and this may possibly lead to a longer treatment delay. Together, such findings imply that poor MHL could contribute to the treatment gap among individuals with mental illness.

A review of the age of onset of mental illness found that most mental illnesses typically emerge during adolescence or early adulthood. However, the afflicted individual usually does not seek treatment until years later (12). This delay in help-seeking for young people can have a deleterious impact on their adult life, as it causes impediments to their emotional well-being and social development (13). Consequently, this might result in substance abuse, lower quality of adult life and even premature death of the individual (14). Consistent with the review by de Girolamo et al. (12), young adulthood has also been identified by Vaingankar et al. (15) to be the likely period for the development of mental illnesses in Singapore, where the median age of onset for common mental disorders is 22, and the majority did not seek treatment within the first year of onset (15). In addition, those with higher education were also less likely to seek treatment for their mental health issues, as evinced by the findings from the SMHS 2016 (10). University students in Singapore are therefore, an important population when considering the reduction of the mental illness treatment gap, and a potentially viable strategy to do so would be to increase the MHL among this particular subpopulation. On top of improving the young person's help-seeking capabilities, such an approach is also likely to enhance their ability to recognize distress among peers and extend appropriate help.

According to Kutcher et al., “MHL interventions need to be contextually developed and developmentally appropriate.” Thus, MHL interventions needs to be framed in appropriate lifespan domains and delivered in the context (i.e., educational settings) relevant to the target audience (i.e., students) (16). Therefore, this study aims to assess whether an education and contact-based anti-stigma intervention—which was evinced to be effective at decreasing stigma and improving community attitudes toward depression (17, 18)—would be effective in improving university students' recognition of depression [the most pervasive mental health condition amongst young adults in Singapore (19)] and their help-seeking preference, given that correct recognition and treatment beliefs are important in increasing appropriate help-seeking (6). Further, this study also examines whether the improvements gained from the intervention in terms of recognition and treatment beliefs would be sustained at 3-months post-intervention.

## Methods

### Participants and Procedures

Using a convenience sampling strategy, a total of 390 students from a university in Singapore were recruited for the study from October 2018 to April 2019. Data was collected as part of the Advancing Research Toward Eliminating Mental Illness Stigma (ARTEMIS) study, a repeated measures study which evaluated the effectiveness of an anti-stigma intervention.

The intervention began with a lecture, delivered by a female mental health professional who has a Masters in Clinical Psychology, that imparted knowledge on depression such as the prevalence, causes and available treatment options. The lecture also comprised a video by the WHO titled “I had a black dog, his name was depression.” The video was narrated by a male voice and can be accessed on youtube, via the hyperlink https://www.youtube.com/watch?v=XiCrniLQGYc. The lecture was followed by a sharing session by a person with lived experience of mental illness on her journey to recovery. The person with lived experience of mental illness was a female in her 20s with a diploma in communications, and was an ambassador of the IMH's Community Health Assessment Team (CHAT). The person with lived experience was chosen because of her relatively young age, which was close to that of the target audience, and because of her experience as a CHAT ambassador which made her an eloquent presenter of such topics. Concluding the intervention was a question-and-answer (QnA) session led by a consultant psychiatrist and a mental health research expert. On average, the intervention spanned 50 min.

Participants of this study had to complete 3 sets of identical questionnaires; prior to commencement of the intervention (baseline), immediately after the intervention (time-point 2), and 3-months from date of intervention (time-point 3). More details of the ARTEMIS study design can be found in previously published articles (18, 20).

Participants were between 18 and 35 years of age and studying at the university at the point of recruitment, as well as literate in English. Written informed consent was obtained from all participants, and parental consent was obtained from those below 21 years of age. This study was approved by the relevant institutional ethics committee, the Domain Specific Review Board of National Healthcare Group in Singapore.

### Instruments

#### Sociodemographic Questionnaire

Sociodemographic information such as age, gender, ethnicity, and year of study were collected from participants using a self-administered questionnaire. In the same questionnaire, participants were also asked to indicate their prior experience in the mental health field if any, and whether they know any close friends or family members with mental illness.

#### Recognition and Help-Seeking Beliefs

Participants' recognition of depression and help-seeking beliefs were both assessed using a vignette approach similar to that of earlier studies (21, 22). However, for this study, participants were given only the vignette which describes a man with symptoms of depression (see Appendix A). The vignette was accompanied by two open-ended questions “What do you think Adam is suffering from?” and “Who do you think Adam should seek help from?” which assessed recognition of depression and help-seeking preference respectively.”

### Coding

Two coders of the study team (MS and GT) independently coded the open text responses for both questions, and the coding for responses were then juxtaposed to ensure consistency. In the event of disagreement over ambiguous response, the two coders would discuss before coming to a consensus on the coding.

For the coding on recognition, the coders took reference from an earlier study that employed a vignette approach among a sample of local medical students (23). Responses were first coded as either “correct” or “incorrect” recognition. If responses contained at least one of any variants of the term “depressive” or “depression” in their answers, they were coded as “correct,” and other responses were coded as “incorrect.” For the “incorrect” responses, they were further classified into different categories. Responses that pertained to symptoms of depression such as insomnia were classified under “Disorder-specific Symptoms.” For responses that mentioned other mental illnesses such as anxiety, PTSD, adjustment disorder or if they simply mentioned mental illness, they were classified under “Mislabeled.” “Not an Illness” comprised of responses that alluded to Adam not having a mental illness, such as “passing away of a loved one,” “disappointment,” “work pressure” or “overstressed,” “social withdrawal,” “family issues,” and “not enough confidence in self.” Lastly, unsure responses were classified as “Unsure.”

For the coding on the “Who do you think Adam should seek help from” question, coders took reference from an earlier nationwide mental health literacy study (11). When the response contained multiple sources of help, only the first response was coded. The responses were coded as follows, (i) “psychiatrist,” (ii) “psychologist,” (iii) “counselors,” (iv) “seek help from IMH (Institute of Mental Health, the only tertiary psychiatric hospital in Singapore),” (v) “unspecified mental health professional,” (vi) “unspecified health professional,” (vii) “family physician or GP,” (viii) “family and friends,” (ix) “workplace” and (x) “others.” For responses such as “therapist,” “mental health professional,” or “mental health expert” where the exact mental health professional's role was not explicitly described, they were classified under “unspecified mental health professional.” For responses such as “professional,” “professional help,” “clinic” or a “a certified medical professional,” where the exact form of professional help was not explicitly stated, they were classified under “unspecified health professional.” Responses such as “God,” “social worker,” “enlightened being” and “anyone,” which were endorsed by <3% of participants were classified as “others.” These responses were then re-classified into two groups, namely, “Psychiatric Help” (i–vii) and “Non-psychiatric help” (viii–x).

### Statistical Analysis

All statistical analyses were performed using IBM SPSS, version 23.0. For descriptive statistics, frequencies and percentages were presented for categorical variables while means and standard deviations were presented for continuous variables. As there was an under-representation of students of the Malay, Indian and other ethnicity, they were subsumed into a single category (non-Chinese) and compared against Chinese ethnicity in the analysis.

To investigate the effects of intervention on recognition of depression, pairwise comparison between pre-intervention and post-intervention were performed using general estimating equations (GEE). Recognition of vignette (correct vs. incorrect) was set as the dependent variable, with time-point (1 = pre-intervention, 2 = post-intervention) included in the GEE as both a fixed effect and within-subject variable to account for both overall and individual variations in recognition. GEE was also performed to compare recognition of vignette between pre-intervention and 3-months follow-up (time-point 3), and post-intervention and 3-months follow-up to assess lasting impacts of intervention.

Likewise, 3 series of GEE comparing between the aforementioned time-points were also performed for help-seeking beliefs (psychiatric vs. non-psychiatric help), with both time-point and recognition of vignettes included as fixed effect and within-subject variables.

Sociodemographic variables such as age, gender, ethnicity, past experience in mental health field, and having a close friend/family with mental illness were included in all the GEE analyses as time-invarying covariates. Significant predictors were then tested for interaction effects with time-point. To account for the attrition at time-point 3 (there were some students who dropped out), the GEE pairwise comparisons involving time-point 3 were handled with listwise deletion. Statistical significance for all analyses was set at alpha level of *p* < 0.05 using two-tailed tests.

## Results

### Descriptive Analysis

Sample characteristics of participants are displayed in Table 1. There were 390 students for time-point 1 and 2. Mean age of participants was 22.3 ± 2.2 years. The majority were females (60.3%), Chinese (82.8%), and had no past experience in a mental health field (77.2%). Slightly less than half of participants had a family or close friend with mental illness (42.6%). There was some attrition at the 3-months follow-up, and total number of participants was 324, with the majority being female (60.8%), Chinese (84.0%), no past experience in mental health field (76.2%). Slightly less than half of the 324 participants knew a close friend or family with mental illness (41.4%), and their mean age was 22.2 ± 2.2 years.

**Table 1:** Sociodemographic characteristics of the sample.

	**Time-point 1 and 2 (*****n*** **= 390)**		**Time-point 3 (*****n*** **= 324)**	
	***n***	**%**	***n***	**%**
**Gender**				
Female	235	60.3	197	60.8
Male	155	39.7	127	39.2
**Ethnicity**				
Chinese	323	82.8	272	84.0
Others	67	17.2	52	16.0
**Family or friends with mental illness**				
Yes	166	42.6	134	41.4
No	224	57.4	190	58.6
**Past experience in mental health field**				
Yes	86	22.1	75	23.1
No	301	77.2	247	76.2
	Mean	S.D.	Mean	S.D.
Age (in years)	22.38	2.26	22.25	2.24

### Recognition of Depression

Table 2 shows the percentage of the participants' responses with regards to the correct recognition of diagnosis (at baseline, post-intervention, and follow-up; and the correct recognition were 90.5, 96.4, and 96.9% respectively). In relation to the incorrect responses, the most common was “Not an Illness” (4.4%) for baseline, “Mislabeled” (2.6%) for post-intervention and “Not an Illness” (1.5%) for follow-up.

**Table 2:** Student's description of Adam's problem in pre (time-point 1) and post (time-point 2) intervention as well as 3-months (time-point 3) from intervention.

	**Time-point 1 (*****n*** **= 390)**				**Time-point 2 (*****n*** **= 390)**				**Time-point 3 (*****n*** **= 324)**			
**Recognition**	**Correct**		**Incorrect**		**Correct**		**Incorrect**		**Correct**		**Incorrect**	
	***n***	***%***	***n***	***%***	***n***	***%***	***n***	***%***	***n***	***%***	***n***	***%***
	**353**	**90.5**	**37**	**9.5**	**374**	**96.4**	**14**	**3.6**	**314**	**96.9**	**10**	**3.1**
**Incorrect Classifications**			***n***	***%***			***n***	***%***			***n***	***%***
Disorder Specific Symptoms			7	1.8			1	0.3			2	0.6
Mislabeled			10	2.6			9	2.3			2	0.6
Not an Illness			17	4.4			4	1			5	1.5
Unsure or Don't Know			3	0.8			0	0			1	0.3

### Help-Seeking Beliefs

Table 3 displays the percentage of students endorsing each category of help-seeking options at baseline, post-intervention and follow-up. At baseline, slightly more than half of all students endorsed seeking “psychiatric help” (58.3%), with “counselor” (22.7%) being the most mentioned sub-category. At post-intervention, the percentage of responses endorsing “psychiatric help” increased to 77.2%, with “psychiatrist” (30.0%) being the most mentioned option of “psychiatric help.” At 3-month follow-up, the percentage of “Psychiatric help” endorsement dropped to 64.5%, and “counselors” (25.0%) was the most mentioned. Across all 3 time-points, “family and friends” was the most endorsed source of “Non-psychiatric help.”

**Table 3:** Students' belief about where Adam should seek help from in pre (time-point 1) and post (time-point 2) intervention as well as 3-months (time-point 3) from intervention.

	**Time-point 1 (*****n*** **= 390)**				**Time-point 2 (*****n*** **= 390)**				**Time-point 3 (*****n*** **= 324)**			
**Type of help**	**Psychiatric**		**Non-Psychiatric**		**Psychiatric**		**Non-Psychiatric**		**Psychiatric**		**Non-Psychiatric**	
	***n***	***%***	***n***	***%***	***n***	***%***	***n***	***%***	***n***	***%***	***n***	***%***
	218	58.3	156	41.7	288	77.2	85	22.8	209	64.5	115	35.5
	***n***	***%***			***n***	***%***			***n***	***%***		
Counselors	85	22.7			107	28.7			81	25.0		
Psychologists	45	12.0			40	10.7			32	9.9		
Psychiatrists	57	15.2			112	30.0			62	19.1		
Seek Help from IMH^*^	8	2.1			22	5.9			8	2.5		
Unspecified Mental Health Professionals	23	6.1			7	1.9			26	8.0		
			***n***	***%***			***n***	***%***			***n***	***%***
Unspecified Health Professionals			19	5.1			25	6.7			27	8.3
Family Physician or GP			25	6.7			10	2.7			10	3.1
Family and Friends			97	25.9			43	11.5			65	20.1
Workplace			5	1.3			2	0.5			5	1.5
Others			10	2.7			5	1.3			8	2.5

* *IMH refers to Institute of Mental Health, the only tertiary mental health institute in Singapore*.

### GEE Analysis for Recognition of Depression

GEE analysis revealed having a close friend or family member to be a significant predictor of recognition of vignette across all 3 pairwise comparison of time-points, and that students at time-points 2 and 3 when compared to time-point 1 were more likely to correctly recognize depression from the vignette (see Table 4). Pairwise comparison between time-points 2 and 3 however showed no significant difference. There was no significant interaction between time-points and any of the time-invarying variables, and thus the analysis was not included in the final model.

**Table 4:** Impact of ARTEMIS on recognition after controlling for co-variates using Generalized Estimating Equation (incorrect recognition set as reference group).

**Time-point 1 and 2 (*****n*** **= 390)**				**Time-point 2 and 3 (*****n*** **= 324)**				**Time-point 1 and 3 (*****n*** **= 324)**			
	**OR**	**95% CI**	***P*-Value**		**OR**	**95% CI**	***P*-Value**		**OR**	**95% CI**	***P*-Value**
Age	0.953	0.824 to 1.101	0.510	Age	0.837	0.638 to 1.097	0.197	Age	0.931	0.750 to 1.157	0.521
Gender				Gender				Gender			
Male	0.656	0.296 to 1.457	0.301	Male	1.866	0.462 to 7.537	0.381	Male	0.732	0.265 to 2.021	0.547
Female	Ref			Female	Ref		.	Female	Ref		.
Ethnicity				Ethnicity				Ethnicity			
Non-Chinese	0.521	0.243 to 1.117	0.094	Non-Chinese	0.705	0.210 to 2.360	0.570	Non-Chinese	0.879	0.279 to 2.766	0.825
Chinese	Ref			Chinese	Ref		.	Chinese	Ref		.
Close friend/family with mental illness				Close friend/family with mental illness				Close friend/family with mental illness			
Yes	2.161	1.010 to 4.624	0.047^*^	Yes	6.412	1.303 to 31.564	0.022^*^	Yes	4.026	1.422 to 11.396	0.009^**^
No	Ref		.	No	Ref		.	No	Ref		.
Past experience in mental health field				Past experience in mental health field				Past experience in mental health field			
Yes	1.846	0.827 to 7.569	0.287	Yes	2.844	0.444 to 18.236	0.270	Yes	2.733	0.746 to 10.010	0.129
No	Ref		.	No	Ref		.	No	Ref		.
Time-Point				Time-Point				Time-Point			
2	2.816	1.667 to 4.755	<0.001^**^	3	0.898	0.409 to 1.972	0.788	3	2.949	1.610 to 5.400	<0.001^**^
1	Ref	.	.	2	Ref	.	.	1	Ref	.	.

* *p-value significant at <0.05*.

** *p-value significant at <0.01, Time-point 1: Pre-intervention, Time-point 2: Post-intervention, Time-point 3: 3-months follow-up*.

*Values in bold denotes significance <0.05 or <0.01*.

### GEE Analysis for Help-Seeking Beliefs

Table 5 shows the GEE results with Help-Beliefs as the dependent variable. Students who were able to correctly recognize the vignette, as compared to those who not, were significantly more likely to recommend psychiatric help over non-psychiatric help (O.R = 2.146, α = 0.001) in the pairwise comparison between time-point 1 and 2, and time-point 1 and 3 Students at time-point 2 when compared to time-point 1 were significantly more likely to endorse seeking help from psychiatric help options (O.R = 2.320, α <0.001). While there was no significant difference between time-point 3 and 1, students at time-point 3 were significantly less likely to endorse psychiatric help options than at time-point 2. There was no significant interaction between time-points and recognition or any of the time-invarying variables, and thus the analysis was not included in the final model.

**Table 5:** Impact of ARTEMIS on help-seeking preferences after controlling for co-variates using Generalized Estimating Equation (non-psychiatric help set as reference group).

**Time-point 1 and 2 (*****n*** **= 390)**				**Time-point 2 and 3 (*****n*** **= 324)**				**Time-point 1 and 3 (*****n*** **= 324)**			
	**OR**	**95% CI**	***P*-Value**		**OR**	**95% CI**	***P*-Value**		**OR**	**95% CI**	***P*-Value**
Age	0.992	0.907 to 1.085	0.864	Age	0.985	0.896 to 1.083	0.751	Age	0.987	0.906 to 1.075	0.760
Gender				Gender				Gender			
Male	0.788	0.520 to 1.192	0.259	Male	0.923	0.589 to 1.446	0.725	Male	0.811	0.527 to 1.246	0.338
Female	Ref		.	Female	Ref		.	Female	Ref		.
Ethnicity				Ethnicity				Ethnicity			
Non-Chinese	0.639	0.629 to 1.381	0.067	Non-Chinese	0.886	0.505 to 1.554	0.673	Non-Chinese	0.917	0.547 to 1.538	0.743
Chinese	Ref		.	Chinese	Ref		.	Chinese	Ref		.
Close friend/family with mental illness				Close friend/family with mental illness				Close friend/family with mental illness			
Yes	0.932	0.629 to 1.381	0.726	Yes	0.898	0.594 to 1.356	0.608	Yes	0.965	0.645 to 1.445	0.864
No	Ref		.	No	Ref		.	No	Ref		.
Past experience in mental health field				Past experience in mental health field				Past experience in mental health field			
Yes	1.088	0.693 to 1.708	0.713	Yes	0.895	0.552 to 1.452	0.653	Yes	0.857	0.530 to 1.384	0.528
No	Ref		.	No	Ref		.	No	Ref		.
Recognition of depression				Recognition of depression				Recognition of depression			
Correct	2.178	1.328 to 3.573	0.002^**^	Correct	1.258	0.476 to 3.327	0.644	Correct	3.217	1.622 to 6.379	0.001^*^
Incorrect	Ref		.	Incorrect	Ref		.	Incorrect	Ref		.
Time-Point				Time-Point				Time-Point			
2	2.306	1.795 to 2.963	<0.002^**^	3	0.496	0.366 to 0.670	<0.001^**^	3	1.214	0.943 to 1.564	0.133
1	Ref	.	.	2	Ref	.	.	1	Ref	.	.

*Time-point 1: Pre-intervention*.

* *p-value significant at <0.05*.

*Time-point 2: Post-intervention*.

** *p-value significant at <0.01, Time-point 3: 3-months follow-up*.

*Values in bold denotes significance <0.05 or <0.01*.

### *Post-hoc* Analyses

Due to attrition, GEE analyses involving time-point 3 had a smaller sample size compared to the GEE analysis between time-point 1 and 2. As such, it is possible that the fluctuation in assessment may have influenced the results of GEE analyses involving time-point 3. Hence, a 2x2 chi-square analysis were performed comparing the recognition and help-seeking preferences at time-point 1 and 2 between students who dropped out and students who continued participating in the study. The analysis revealed no significant differences, indicating that the results of the GEE analysis are unlikely to be due to attrition.

As the recognition of depression was already high at time-point 1, given that having a close friend/family member with MI was also a significant predictor of correct recognition, it is possible that the finding of students being more likely to recognize depression at time-point 2 compared to time-point 1 may not be due to the effectiveness of the intervention. Hence, an additional 2x2 chi-square was run to test whether participants with close friend or family member were more likely to recognize depression. The analysis found no significant differences between recognition and having close friend/family member with MI, indicating that the significant increase in correct recognition at time-point 2 as compared to time-point 1 is more likely due to the effects of the intervention. The results for these analyses can be found in Table 6.

**Table 6:** *Post-hoc* sensitivity analyses of: (i) recognition of depression between participants who dropped out and those who didn't at pre-intervention (ii) recognition of depression between participants who dropped out and those who didn't at post-intervention (iii) recognition of depression between participants with close friend/family with MI and those without at post-intervention (iv) help-seeking preferences between participants between participants who dropped out and those who didn't at pre-intervention (v) help-seeking preferences between participants between participants who dropped out and those who didn't at post-intervention.

**Attrition**				**Total**	***p*-value**
**i) Recognition of depression**					
		**Participants who stayed**	**Participants who dropped out at 3-months**		
Pre	Correct	297	10	324	0.104
	Incorrect	56	27	66	
Total	353	37	390		
**ii) Recognition of depression**					
		**Participants who stayed**	**Participants who dropped out at 3-months**		
Post	Correct	314	9	323	0.067
	Incorrect	60	5	65	
Total	374	14	388		
**iii) Recognition of depression**					
		**Yes**	**No**		
Post	Correct	162	212	374	0.410
	Incorrect	4	10	14	
Total	166	222	388		
**iv) Help-seeking Preferences**					
		**Participants who stayed**	**Participants who stayed**		
Pre	Psychiatric Help	35	183	218	0.578
	Non-psychiatric Help	29	127	156	
Total	64	310	374		
**v) Help-seeking Preferences**					
		**Participants who stayed**	**Participants who stayed**		
Post	Psychiatric Help	245	43	288	0.135
	Non-psychiatric Help	66	19	85	
Total	311	62	373		

Two *post-hoc* power analyses was conducted using PS Power and Sample Size Calculator (24). The Type I error probability associated with this test of this null hypothesis is 0.05. Our data indicate that the proportion of correct recognition at baseline was 90.5% and the correlation coefficient between baseline and post-test was 0.367. If the true odds ratio is 2.816, we will be able to reject the null hypothesis that this odds ratio equals 1 with probability (power) of 83.3%. For help-seeking preference, our data indicate that the proportion of help-seeking preference at baseline was 58.3% and the correlation coefficient between baseline and post-test was 0.415. If the true odds ratio is 2.306, we will be able to reject the null hypothesis that this odds ratio equals 1 with probability (power) of 99.1%.

## Discussion

To our knowledge, this is the first study that evaluated the effects of an educational intervention in conjunction with contact-based intervention on recognition of depression and help-seeking beliefs among university students immediately post intervention and at 3-months after the intervention.

Correct recognition of depression at baseline in this study was high (90.5%), which is only slightly lower than that of a previous study which sampled medical students (93.0%) (23). On the other hand, the rate of recognition among our sample is slightly higher than that of nursing students (85.0%) (25), considerably higher than that of the previous nationwide MHL study which sampled the general population in Singapore (55.2%) (21), and also considerably higher than that of a study in England which sampled 3,004 young adults between 16 and 24 years (61.4%) (26). However, unlike the previous nationwide study by Chong et al. (21) which consisted of adults from various age groups and Klineberg et al.'s (26) study of young adults, gender was not a significant predictor for recognition of depression in this study. The lack of significant difference between gender in recognizing depression in our study is similar to studies by Seow et al. (25) and Picco et al. (23) which also employed student participants who mostly fall within the emerging adulthood range. Collectively, the findings from Seow et al. (25), Picco et al. (23) and our study suggest that there is a better understanding and awareness of mental health issues among the current student population, and that this phenomenon is generalizable across both genders, which possibly mediated the effects that gender may have toward recognition of depression.

Knowing a family or friend with mental illness was found to be significantly associated with correct recognition of depression in all 3 analysis of GEE, and this is consistent with the finding from another study, which reported that having previous contact with a person with mental illness (PMI) positively influenced the recognition of depression (27). This corroborates the evidence from literature which suggests that intergroup contact such as exposure to, or experience in interacting with someone with mental illness results in improved mental health literacy (28, 29). Interestingly, having past experience in the mental health field was not a significant predictor of recognition, even though these participants were also likely to have had contact with PMI. There are a few plausible explanations for this phenomenon. As this study did not take into account the duration of students' past experience in the mental health field as well as the specific kind of experience, it is possible that some students' past experience may be a one-time occurrence (i.e., mandatory school community activities) or that their past involvement in the mental health scene may have been one where there was very limited interaction with PMI. In this regard, the student's ability to recognize mental illnesses is unlikely to improve from their brief experience in the mental health field. In contrast, one would arguably have had more instances of social contact/interaction with PMI and thus better recognition toward mental illness if they have a close friend or family member with mental illness. Furthermore, having a friend or family member with mental illness may prompt an individual to find out more about mental illness, therefore increasing their MHL and potentially their ability to recognize mental illness as well.

Despite the correct recognition being high at baseline (90.5%), there were still significant improvements at post-intervention. Furthermore, participants at 3-month follow-up were still significantly more likely to correctly recognize the vignette as compared to baseline, indicating the lasting impact of the intervention on recognition at 3-months. However, given the lack of a control group, there is a need to acknowledge that there could be an alternative explanation for this finding, considering that the recognition of depression was high from the onset and across all 3 time-points. It is possible that students having undertaken the pre-test, might have been primed to giving the correct answer in the post-test and during the subsequent 3-months follow-up. Nonetheless, it is possible that the improvement in recognition of depression at post-intervention might indeed be attributed to the effects of the intervention; because student's knowledge of depression did improve after the intervention as evidenced in an earlier published paper (18), and the findings from Australia's Beyond Blue campaign suggests that an increase in knowledge of depression can improve recognition (30). Regardless, it is recommended for future studies to include a control group, in order to further validate the effectiveness of the intervention in improving recognition of depression.

With regard to help-seeking beliefs at baseline, the proportion of those who endorsed help-seeking from family and friends (25.9%) is very similar to the study by Picco et al. (22) of medical and nursing students' help-seeking beliefs study, and considerably lower compared to that of the general population (11). In addition, compared to the nationwide study, the two studies reported higher preference of seeking help from options classified under “Psychiatric help” in this paper. This perhaps reflects better MHL among the current younger cohort in Singapore, as MHL consists also of the knowledge of available professional help and attitudes that promote recognition and appropriate help-seeking (1). The findings from another study (31) which found that younger people had better knowledge on recognition and treatment of depression also lends credence to this explanation.

Seeking help from a counselor (22.7%) was the most commonly endorsed form of “Psychiatric help' in this study at baseline, whereas seeing a psychiatrist was the most endorsed (39.5%) form in Picco et al.'s (22) study. This difference is probably due to the different type of students who were surveyed: in that the participants Picco et al.'s (22) study were medical students whereas the participants in our sample were non-medical university students from a range of disciplines. Moreover, the greater preference to seek help from a counselor among our sample may be tied to the fact that students are more aware of counselors as a help-seeking option, given that the university provides an on-campus student counseling service.

The results of the GEE for help-seeking preferences between time-point 1 and 2 and time-point 1 and 3, found that the ability to recognize depression is associated with increased likelihood of seeking psychiatric help. This is consistent with the study by Wright et al. (32), which found that among various predictor variables, correct labeling of a disorder (depression and psychosis vignettes in this case) was the variable most frequently associated with appropriate treatment and help-seeking choices (32). This further reinforces the influence that recognition has on seeking evidence-based mental health care as posited by Jorm (33). A rather inexplicable finding was that recognition was not found to be a significant predictor for help-seeking preference in the comparison between time-point 2 and time-point 3. This could be due to ceiling effect, as the correct recognition of depression at both time-point 2 (97.25) and 3 (96.9%) were very high, and thus the lack of significance could be due to low power because the sample size for incorrect recognition in this analysis was too small.

Another significant finding from this analysis was the increased likelihood to endorse psychiatric help at time-point 2 when compared to time-point 1, suggesting that the intervention positively influenced student's preference to seek psychiatric help. This could be attributed to student's increase in knowledge of available help-seeking options. In addition, the respondents had the benefit of listening to a psychiatrist during the question-and-answer section following the intervention. Alternatively, it may be that the direct contact with a person with lived experience of mental illness, which was part of the intervention, helped alleviate participants' stigma toward mental illness, and in turn reduced student's stigma toward seeking psychological help. This is supported by Hantzi et al.'s study (34) which found that when there are lesser negative beliefs about mental illness, the self-stigma for seeking psychological help is reduced, while positive help-seeking attitudes are increased (34).

However, unlike recognition, there were no significant differences for help-seeking beliefs at time-point 3 vs. time-point 1, indicating that the gains from the intervention were not sustained at time-point 3. In fact, pairwise comparison revealed a significant decrease in likelihood of endorsing psychiatric help from time-point 2 to time-point 3. It is possible that the gains from recognition were more likely to be sustained because recognition of an illness is very much based on knowing the signs and symptoms of the illness, while help-seeking preference is more complex. On top of knowing where to seek appropriate help from and the belief in its effectiveness, help-seeking preference possibly also involves multiple factors which interact with each other such as stigma, accessibility to mental health services, and whether one has the time and capacity to utilize these mental health services. In particular, there could be more stigma attached to consulting a psychiatrist, who at the same time, may be perceived to be less accessible than on-campus counseling services which the university provides at no charge.

An alternate postulation for the observed trend in help-seeking beliefs at different time points is that the intervention may have evoked positive emotions among students toward the psychiatric profession and the utilization of mental health services. A study on female university students' readiness to seek help from a professional helper—in this case a counselor—was associated with anticipation of positive emotions through help-seeking, and these emotions may be influenced by helper's characteristics (35). Likewise, in our study, anticipated positive emotions of help-seeking may have been evoked among students during the Q&A section with a senior psychiatrist from IMH, which likely contributed to the increase in endorsement of psychiatric help immediately after the intervention. Furthermore, the sharing of lived experience with mental illness by the person who had past history of it, and who is also a CHAT Ambassador with IMH, probably reinforced the importance of seeking psychological help. Notably, endorsement in seeking help from a psychiatrist and IMH had both approximately doubled from time-point 1 to time-point 2, further lending credence to our proposed postulation.

In addition, the sharing from the person with lived-experience about her journey to recovery might have, evoked some positive emotions among students, which promoted their willingness to seek psychiatric help. This postulation is perhaps supported by a recent study which found a plausible causal relationship between experiencing a story-based elevation induction and increased help-seeking intentions (36); and elevation in this study refers to a warm and uplifting emotion that is posited to enhance people's outlook on humanity, increase their confidence in recovering when treatment is sought, as well as allowing them to feel less likely to be judged. Hence, the infusion of positive feelings toward help-seeking may have resulted in the significant increase in endorsement of “Psychiatric help” at time-point 2. However, as it is highly unlikely for these positive feelings induced by the intervention to be sustained for 3-months without waning, this perhaps accounts for the observed trend in help-seeking beliefs across time-points.

## Limitations

There are a couple of limitations in this study that needs to be acknowledged. Firstly, as the study used convenience sampling, the results may not be generalizable as there may be some self-selection bias. There is a possibility that students who volunteered for this study are generally more empathetic toward those with mental health issues or had personal interest in participating in the study. As such, future studies could replicate the current one across other non-self-selected samples, perhaps by integrating the ARTEMIS as part of a curriculum or during students' orientation or other student activities, in order to evaluate the generalizability of results.

Secondly, some studies have posited that young people have a tendency to over-identify depression (21, 31, 32). In which case, the high rate of recognition may be due to this over-identification of mental illness as depression, rather than students actually being well-versed in their understanding of depression. Future research could include vignettes describing other mental illnesses to examine whether this high rate of correct recognition recurs for depression.

Additionally, as this is a single-arm intervention study, the lack of a control group for comparison leaves the observed findings open to other explanations, especially for the recognition of depression. Moreover, the sample size for this study is relatively small. As such, it is recommended for future replica studies to include a control group and increase the sample size in order to further validate the effectiveness of the intervention.

Social desirability bias in the way students answered, is another possible limitation of the study even though they were assured confidentiality, especially with regards to the question on help-seeking, given the direct contact with a psychiatrist during the Q&A section of the intervention. Furthermore, as this is was the first study done in Singapore, there were no earlier studies to compare to for consistency of results. Lastly, although it is recommended for psychoeducation programs that combat mental illness stigma to involve multiple sessions, the current study had only one intervention session given the resource and time constraints. In spite of such limitations, the study presents an early attempt to examine the impact of an anti-stigma intervention on Singapore's university students' recognition of depression as well as their help-seeking preferences.

## Conclusion

This study elucidated the impacts of an anti-stigma intervention on university students' recognition of depression as well as their help-seeking preference. Findings from this study highlighted the efficacy of this knowledge-contact-based intervention in the immediate improvement of both aspects at post-intervention. However, while the benefits on recognition of depression is more enduring, it is more transient for help-seeking beliefs. Therefore, to improve the long-term effectiveness of this intervention on help-seeking beliefs, it is recommended for this intervention to be augmented with follow-up booster sessions so as to maintain the effects of the intervention.

## Data Availability Statement

The datasets presented in this article are not readily available and readers who wish to gain access to the data will have to write to the senior author MS to request access. Requests to access the datasets should be directed to Prof. Mythily Subramaniam, mythily@imh.com.sg.

## Ethics Statement

The studies involving human participants were reviewed and approved by the relevant institutional review board (National Healthcare Group, Domain Specific Review Board). The patients/participants provided their written informed consent to participate in this study.

## Author Contributions

GT was responsible for writing the manuscript, conducting the fieldwork, statistical analysis, and coding with MS. SS, CG, WO, and ES conducted the fieldwork and contributed to study's design. EA conducted power analysis and offered input to study's design. JL contributed to statistical analysis of data. SC, MS, and KK contributed to study's design and supervised the overall study. All authors provided intellectual input to the manuscript and have given their final approval of the version to be published.

## Conflict of Interest

The authors declare that the research was conducted in the absence of any commercial or financial relationships that could be construed as a potential conflict of interest.

## References

[1] Jorm AF Korten AE Jacomb PA Christensen H Rodgers B Pollitt P. “Mental health literacy”: a survey of the public's ability to recognise mental disorders and their beliefs about the effectiveness of treatment. Med J Aust. (1997) 166:182–6. 10.5694/j.1326-5377.1997.tb140071.x 9066546

[2] Smith CL Shochet IM. The impact of mental health literacy on help-seeking intentions: results of a pilot study with first year psychology students. Int J Ment Health Promot. (2011) 13:14–20. 10.1080/14623730.2011.9715652

[3] Tonsing KN. A review of mental health literacy in Singapore. Soc Work Health Care. (2018) 57:27–47. 10.1080/00981389.2017.1383335 28976296

[4] Waldmann T Staiger T Oexle N Rüsch N. Mental health literacy and help-seeking among unemployed people with mental health problems. J Ment Health. (2019) 29, 270–6. 10.1080/09638237.2019.1581342 30862221

[5] Gulliver A Griffiths KM Christensen H. Perceived barriers and facilitators to mental health help-seeking in young people: a systematic review. BMC Psychiatry. (2010) 10:113. 10.1186/1471-244X-10-113 21192795 PMC3022639

[6] Picco L Abdin E Pang S Vaingankar JA Jeyagurunathan A Chong SA. Association between recognition and help-seeking preferences and stigma towards people with mental illness. Epidemiol Psychiatr Sci. (2018) 27:84–93. 10.1017/S2045796016000998 27927259 PMC6998888

[7] Nsereko JR Kizza D Kigozi F Ssebunnya J Ndyanabangi S Flisher AJ. Stakeholder's perceptions of help-seeking behaviour among people with mental health problems in Uganda. Int J Ment Health Syst. (2011) 5:5. 10.1186/1752-4458-5-5 21314989 PMC3050843

[8] Razali SM Najib MAM. Help-seeking pathways among malay psychiatric patients. Int J Soc Psychiatry. (2000) 46:281–9. 10.1177/002076400004600405 11201349

[9] Bhikha A Farooq S Chaudhry N Naeem F Husain N. Explanatory models of psychosis amongst British South Asians. Asian J Psychiatr. 16:48–54. 10.1016/j.ajp.2015.05.042 26232352

[10] Subramaniam M Abdin E Ajit Vaingankar J Shafie S Choon Chua H Mooi Tan W. Minding the treatment gap: results of the Singapore mental health study. Soc Psychiatry Psychiatr Epidemiol. (2019) 55:1415–24. 10.1007/s00127-019-01748-0 31317246 PMC7578124

[11] Picco L Abdin E Chong SA Pang S Vaingankar JA Sagayadevan V. Beliefs about help seeking for mental disorders: findings from a mental health literacy study in Singapore. Psychiatr Serv. (2016) 67:1246–53. 10.1176/appi.ps.201500442 27524364

[12] de Girolamo G Dagani J Purcell R Cocchi A McGorry PD. Age of onset of mental disorders and use of mental health services: needs, opportunities and obstacles. Epidemiol Psychiatr Sci. (2012) 21:47–57. 10.1017/S2045796011000746 22670412

[13] Kutcher S Venn D. Why youth mental health is so important. Medscape J Med. (2008) 10:275. 19242581 PMC2644010

[14] Divin N Harper P Curran E Corry D Leavey G. Help-seeking measures and their use in adolescents: a systematic review. Adolesc Res Rev. (2018) 3:113–22. 10.1007/s40894-017-0078-8

[15] Vaingankar JA Rekhi G Subramaniam M Abdin E Chong SA. Age of onset of life-time mental disorders and treatment contact. Soc Psychiatry Psychiatr Epidemiol. (2013) 48:835–43. 10.1007/s00127-012-0601-y 23076588

[16] Kutcher S Wei Y Coniglio C. Mental health literacy: past, present, and future. Can J Psychiatry Rev Can Psychiatrie. (2016) 61:154–8. 10.1177/0706743715616609 27254090 PMC4813415

[17] Goh CMJ Shahwan S Lau JH Ong WJ Tan GTH Samari E. Advancing research to eliminate mental illness stigma: an interventional study to improve community attitudes towards depression among University students in Singapore. BMC Psychiatry. (2021) 21:108. 10.1186/s12888-021-03106-4 33602155 PMC7890908

[18] Subramaniam M Shahwan S Abdin E Goh CMJ Ong WJ Tan GTH. Advancing research to eliminate mental illness stigma: the design and evaluation of a single-arm intervention among university students in Singapore. Front Psychol. (2020) 11:1151. 10.3389/fpsyg.2020.01151 32581957 PMC7283943

[19] Subramaniam M Abdin E Vaingankar J Shafie S Yiang Chua B Sambasivam R. Tracking the mental health of a nation: prevalence and correlates of mental disorders in the second Singapore mental health study. Epidemiol Psychiatr Sci. (2019) 29:e29. 10.1017/S2045796019000179 30947763 PMC8061188

[20] Shahwan S Lau JH Goh CMJ Ong WJ Tan GTH Kwok KW. The potential impact of an anti-stigma intervention on mental health help-seeking attitudes among university students. BMC Psychiatry. (2020) 20:562. 10.1186/s12888-020-02960-y 33238951 PMC7690018

[21] Chong SA Abdin E Picco L Pang S Jeyagurunathan A Vaingankar JA. Recognition of mental disorders among a multiracial population in Southeast Asia. BMC Psychiatry. (2016) 16:121. 10.1186/s12888-016-0837-2 27142577 PMC4855433

[22] Picco L Seow E Chua BY Mahendran R Verma S Xie H. Help-seeking beliefs for mental disorders among medical and nursing students. Early Interv Psychiatry. (2019) 13:823–31. 10.1111/eip.12673 29740952 PMC6635751

[23] Picco L Seow E Chua BY Mahendran R Verma S Chong SA. Recognition of mental disorders: findings from a cross-sectional study among medical students in Singapore. BMJ Open. (2017) 7:e019038. 10.1136/bmjopen-2017-019038 29273669 PMC5778286

[24] Dupont WD Plummer WD Jr. Power and sample size calculations. A review and computer program. Control Clin Trials. (1990) 11:116–28. 10.1016/0197-2456(90)90005-M 2161310

[25] Seow LSE Chua BY Xie H Wang J Ong HL Abdin E. Correct recognition and continuum belief of mental disorders in a nursing student population. BMC Psychiatry. (2017) 17:289. 10.1186/s12888-017-1447-3 28784095 PMC5547490

[26] Klineberg E Biddle L Donovan J Gunnell D. Symptom recognition and help seeking for depression in young adults: a vignette study. Soc Psychiatry Psychiatr Epidemiol. (2011) 46:495–505. 10.1007/s00127-010-0214-2 20358174

[27] Lauber C Nordt C Falcato L Rössler W. Do people recognise mentalillness? Eur Arch Psychiatry Clin Neurosci. (2003) 253:248–51. 10.1007/s00406-003-0439-0 14504994

[28] Gronholm PC Henderson C Deb T Thornicroft G. Interventions to reduce discrimination and stigma: the state of the art. Soc Psychiatry Psychiatr Epidemiol. (2017) 52:249–58. 10.1007/s00127-017-1341-9 28144713 PMC5344948

[29] Lauber C Ajdacic-Gross V Fritschi N Stulz N Rössler W. Mental health literacy in an educational elite – an online survey among university students. BMC Public Health. (2005) 5:44–44. 10.1186/1471-2458-5-44 15882465 PMC1156910

[30] Jorm AF Christensen H Griffiths KM. The impact of beyondblue: the national depression initiative on the Australian public's recognition of depression and beliefs about treatments. Aust N Z J Psychiatry. (2005) 39:248–54. 10.1080/j.1440-1614.2005.01561.x 15777361

[31] Farrer L Leach L Griffiths KM Christensen H Jorm AF. Age differences in mental health literacy. BMC Public Health. (2008) 8:125. 10.1186/1471-2458-8-125 18423049 PMC2358892

[32] Wright A Jorm AF Harris MG McGorry PD. What's in a name? Is accurate recognition and labelling of mental disorders by young people associated with better help-seeking and treatment preferences? Social Psychiatry Psychiatr Epidemiol. (2007) 42:244–50. 10.1007/s00127-006-0156-x 17450404

[33] Jorm AF. Mental health literacy. Public knowledge and beliefs about mental disorders. Br J Psychiatry. (2000) 177:396–401. 10.1192/bjp.177.5.396 11059991

[34] Hantzi A Anagnostopoulos F Alexiou E. Attitudes towards seeking psychological help: an integrative model based on contact, essentialist beliefs about mental illness, and stigma. J Clin Psychol Med Settings. (2018) 26:142–57. 10.1007/s10880-018-9573-8 29909480

[35] Yagil D Moshe I. Short communication: helpers' characteristics and problem intimacy as determinants of emotions associated with help-seeking. Couns Psychol Q. (2003) 16:223–8. 10.1080/09515070310001610137

[36] Siegel JT Thomson AL. Positive emotion infusions of elevation and gratitude: increasing help-seeking intentions among people with heightened levels of depressive symptomatology. J Posit Psychol. (2017) 12:509–24. 10.1080/17439760.2016.1221125

