# Supplementary material for: Recognition of Depression and Help-Seeking Preference Among University Students in Singapore: An Evaluation of the Impact of Advancing Research to Eliminate Mental Illness Stigma an Education and Contact Intervention
Source: Front Psychiatry. 2021 May 12;12:582730. doi: 10.3389/fpsyt.2021.582730 (PMC8149891; doi:10.3389/fpsyt.2021.582730)
Supplement: Supplementary file 1 [file Data_Sheet_1.docx]

Appendix A: Vignette and Related Questions

Adam is 30 years old. He has been feeling unusually sad and miserable for the last three weeks. Friends noticed he is no longer his usual cheerful self and he has declined all social gatherings over the past two weeks. Even though he is tired all the time, he has trouble sleeping almost every night. Adam doesn’t feel like eating and has lost weight. He can’t focus on his work and puts off making decisions. Adam feels worthless and even everyday tasks seem too much for him. This has come to the attention of his boss, who is concerned about Adams’s poor work performance.

A2. What do you think Adam is suffering from?
Specify: __________________________________________

A3. Who do you think Adam should seek help from?
Specify: __________________________________________
